# Visualization of X4- and R5-Tropic HIV-1 Viruses Expressing Fluorescent Proteins in Human Endometrial Cells: Application to Tropism Study

**DOI:** 10.1371/journal.pone.0169453

**Published:** 2017-01-06

**Authors:** Rachel Terrasse, Meriam Memmi, Sabine Palle, Leo Heyndrickx, Guido Vanham, Bruno Pozzetto, Thomas Bourlet

**Affiliations:** 1 Groupe Immunité des Muqueuses et Agents Pathogènes EA3064, University of Lyon, Faculté de Médecine Jacques Lisfranc de Saint-Etienne, Saint-Etienne cedex 02, France; 2 Centre de Microscopie Confocale Multiphotonique, Université Jean Monnet, Pôle Optique et Vision, Saint-Etienne cedex 2, France; 3 Virology Unit, Department of Biomedical Sciences, Institute of Tropical Medicine, Antwerp, Belgium; 4 Faculty of Biomedical, Pharmaceutical and Veterinary Sciences, University of Antwerp, Antwerp, Belgium; University of Liverpool Institute of Infection and Global Health, UNITED KINGDOM

## Abstract

Worldwide most HIV infections occur through heterosexual transmission, involving complex interactions of cell-free and cell-associated particles with cells of the female genital tract mucosa. The ability of HIV-1 to “infect” epithelial cells remains poorly understood. To address this question, replicative-competent chimeric constructs expressing fluorescent proteins and harboring the envelope of X4- or R5-tropic HIV-1 strains were used to “infect” endometrial HEC1-A cells. The virus-cell interactions were visualized using confocal microscopy (CM) at various times post infection. Combined with quantification of viral RNA and total HIV DNA in infected cells, the CM pictures suggest that epithelial cells do not support a complete viral replication cycle: X4-tropic viruses are imported into the nucleus in a non-productive way, whereas R5-tropic viruses transit through the cytoplasm without replication and are preferentially transmitted to susceptible activated peripheral blood mononuclear cells. Within the limit of experiments conducted *in vitro* on a continued cell line, these results indicate that the epithelial mucosa may participate to the selection of HIV-1 strains at the mucosal level.

## Introduction

Heterosexual transmission of HIV by semen from infected men to the genital mucosa of uninfected women is the main cause of new infections worldwide. HIV is present in semen as cell-associated virus in non-spermatozoa mononuclear cells as well as cell-free particles. Diverse routes of transmission involving these two forms of viruses through the intact female genital mucosa have been described [1–10]. One model suggests that cell-free particles pass through the epithelial cells by transcytosis [1,11]. Alternatively, seminal mononuclear cell-associated viruses may transmigrate through the female genital tract [12,13]. Dendritic cells and Langerhans cells were also shown to play a key role in the transport of virions across the mucosa, as recently reviewed in [14]. However, many questions persist on the relevance of these different mechanisms for HIV *in vivo* transmission, on the respective role of mono- or multi-layered epithelium for modulating this passage and on the form of virus being preferentially transmitted. The susceptibility of epithelial cells from various tissues to HIV infection and their participation in viral transmission through the epithelial barriers is also largely debated (reviewed recently in [10] and [15]). In the era of new prevention strategies, including pre-exposure prophylaxis, microbicides and treatment as prevention, understanding these mechanisms will help to develop more efficient preventive weapons against HIV [3,16].

In order to visualize virus-cell interactions, several authors have developed different types of virological tools, such as luminescent single-cycle pseudoviruses, replication competent fluorescent viruses and directly-tagged viral particles [9,17–23]. On the other hand, various *in vivo*, *ex vivo* and *in vitro* models have been described to study mucosal HIV transmission. Although *in vivo* models are undoubtedly the most relevant ones [24], they are too expensive and time-consuming for large numbers of experiments. *Ex vivo* models based on cervicovaginal explants respect tissue architecture and its physiological and immunological characteristics; they are of great value for investigating the infection of intact epithelial barriers [5,9,25,26] but are poorly reproducible and may present an artificially-enhanced inflammatory environment. *In vitro* models are constituted of various cell lines including normal human epithelial cells and continuous cell lines [5,7,27–30]. Even though they do not entirely mimic human *in vivo* conditions of HIV crossing, these models have the advantage of being highly reproducible and enable the in-depth study of interactions between virus and genital epithelial cells.

We describe herein an *in vitro* model dedicated to the visualization of virus-epithelial cell interactions. To this end, replicative-competent R5- and X4-tropic chimeric viruses producing fluorescent proteins were constructed and used to infect HEC-1A monolayers. The presence of chimeric viruses in the cells was visualized by confocal microscopy (CM). Together with PCR analysis of HIV-1 DNA, these pictures suggest that epithelial cells do not support a complete viral replication cycle: X4-tropic strains are imported into the nucleus, but no virus production ensues, whereas the R5-tropic virions transit through the cytoplasm, are preferentially transmitted to the other side of the monolayer and can infect activated peripheral blood mononuclear cells (PBMCs).

## Materials and Methods

### Cellular and virological tools

#### Cells

The human endometrial cell line HEC-1A, originating from the American Type Culture Collection (ATCC) [31], was cultured in McCoy’s medium (PAA, Les Mureaux, France) supplemented with 10% of fetal bovine serum (FBS) (HyClone, Thermo Scientific, Courtaboeuf, France) and 1% of antibiotic/antifungal solution (PAA). Three days prior infection, cells were seeded at 1x10^6^ cells/well in 6-well plates on cover glasses (22x22mm #1.5, Menzel-Gläser, Brunswick, Germany) or at 1x10^5^ cells/well in 12-well plates four days prior infection and maintained in complete medium at 37°C under 5% CO_2_.

Epithelial cell surface expression of CD4, CXCR4 and CCR5 molecules was evaluated by flow cytometry analysis (FACS Calibur cytometer, BD biosciences, Le Pont de Claix, France) as previously described [32].

The human embryonic kidney HEK-293 cell line (ATCC) was cultured in DMEM-high glucose medium (PAA) supplemented with 10% of FBS, 1% of antibiotic/antifungal solution and 1% of non-essential amino acids (PAA). Cells were seeded at 2x10^5^ cells/well in 6-well plates, 48 hours prior to transfection experiments and cultured at 37°C under 5% CO_2_.

TZM-bl cells, issued from HeLa human cervical carcinoma cells (ATCC), were cultured in DMEM-high glucose medium supplemented with 10% of FBS and 1% of antibiotic/antifungal solution. Cells were seeded at 2x10^4^ cells/well in 96-well plates, 24 hours prior to viral infection and cultured in complete medium at 37°C under 5% CO_2_.

Human PBMCs were obtained from buffy coats of healthy donors (a gift from the Rhône-Alpes-Auvergne section of the French Blood Bank), purified by gradient density assay using Lymphoprep^TM^ (Abcys, Paris, France), cultured in RPMI 1640 medium (PAA) supplemented with 10% of FBS and 1% of antibiotic/antifungal solution and activated 2 to 3 days before virus infection by addition of 10 μg/ml of phytohaemagglutinin (PHA) and 10 μg/ml of interleukin-2 (IL-2).

#### HIV-1 strains

The HIV-1 BaL (CCR5-tropic virus) and HIV-1 HXB2 (CXCR4-tropic) laboratory strains (NIH AID Research & Reference Reagent Program) were used to construct chimeric viruses.

The HIV-1 Lai laboratory strain (CXCR4-tropic), the HIV-1 BaL strain and two primary isolates, 92UG029 (CXCR4-tropic) and 92US660 (CCR5-tropic), obtained from NIH AIDS Research & Reference Reagent Program, were also used in HEC-1A infection experiments. Cell-free virus was produced after 10 days of infection of IL-2/PHA stimulated PBMCs. Virus titration was performed by p24 ELISA.

#### Quantification of p24 antigen by ELISA

The quantification of p24 antigen was done on supernatants of transfection experiments (chimeric viruses) by an in-house ELISA assay, as previously described [32].The threshold of sensitivity of the test was approximately 100 pg/ml. Samples that scored negative in the in-house ELISA were tested in a more sensitive ELISA from ABL (Rockville USA) with a threshold of 3.1 pg/ml. Optical densities were measured at 450 nm.

#### Amplification and quantification of total HIV DNA and HIV RNA

Epithelial cells were trypsinized, pelleted by centrifugation for 10 min at 430 g and washed prior to DNA or RNA extraction process. Total DNA and RNA were then extracted from 10^6^ cell batches by using the QIAamp DNA minikit (Qiagen, Courtaboeuf, France) and the QIAamp RNA minikit (Qiagen) respectively, according to the manufacturer’s instructions.

The HIV DNA was quantified by real time PCR according to the protocol described by Friedrich et al. [33]. Briefly, an HIV LTR genomic fragment was amplified by the iTaq Fast Supermix (BioRad, Marnes-la-Coquette, France) supplemented with the forward LTR1 *(*5'-GCC TCA ATA AAG CTT GCC TTG A-3') and the reverse LTR2 (5'-TCC ACA CTG ACT AAA AGG GTC TGA-3') primers and the LTR molecular ProHIV probe (5'-FAM-GCG AGT GCC CGT CTG TTG TGT GAC TCT GGT AAC TAG CTC GC-DABCYL-3'). The amplification was performed using an ABI 7500® apparatus (Applied Biosystem, Saint Aubin, France) and the quantification was performed by reference to a standard curve based on serial dilutions of 8E5 cells containing one HIV DNA copy per cell. Results were expressed as number of DNA copies/10^6^ cells.

Extra- and intra-cellular HIV RNA was quantified by the Generic HIV viral load assay (Biocentric, Bandol, France), using the ABI 7500® apparatus. Results were expressed as number of copies/ml. The sensitivity of the assay was of 50 copies of HIV RNA per ml.

### Characteristics of the confocal microscope

The samples were analyzed with a Leica TCS-SP2 confocal scanning laser inverted microscope (Leica-Microsystem, Heidelberg, Germany). The fluorescent markers were excited with a set of three continuous wave lasers delivering monochromatic light at wavelength equal to 488 nm for eGFP excitation, 543 nm for both Alexa Fluor™ 555 and DsRedExpress excitation, and 633 nm for Alexa Fluor™ 647 excitation. An oil immersion objective (HCX PL APO 63X 1.4NA) was used in all experiments. Image stacks were further analyzed in sequential mode, which means that each color was analyzed independently in order to avoid cross-talks of the different signals. Colocalization was defined as the superposition of signals resulting from different lasers at the same place within a stack. The images were processed using ImageJ software [34] to perform average projection of stacks, contrast enhancement and merging of the differential interference contrast (DIC) and fluorescence channels.

### Chimeric viruses

#### Construction

**Fig 1:**
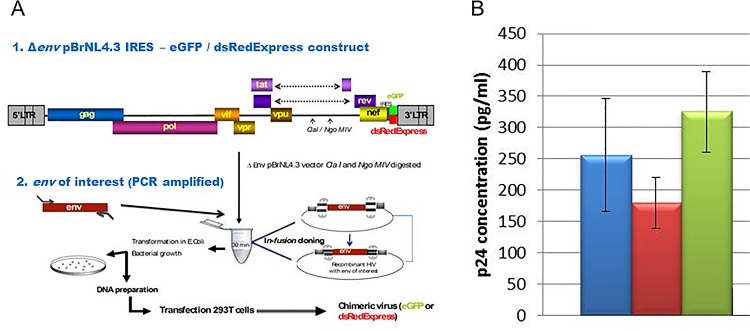
Chimeric viruses used in this study. A. Strategy developed for building the chimeric viruses by inserting a PCR-amplified env gene from HXB2 or BaL HIV-1 laboratory strains to Δenv pBrNL4.3 IRES-eGFP/dsRedExpress constructs (adapted from [36]). B. Quantification of p24 in supernatants of HEK-293 cells after transfection with chimeric viruses: pBrNL4.3-HXB2-eGFP in blue, pBrNL4.3-HXB2-drRedExpress in red, and pBrNL4.3-BaL-eGFP in green. Results are representative of 3 independent transfection experiments and each p24 measurement was performed in duplicate.

Intact recombinant replicative chimeric viruses expressing green (eGFP) or red (dsRedExpress) fluorescent proteins and harboring the envelope of HIV-1 HXB2 (X4-tropic) or BaL (R5-tropic) were produced as previously described [35,36] (Fig 1A). Briefly, 1 μg of plasmid DNA was used to transfect HEK-293, cells cultured in 6-well plates, by using FUGENE 6 Transfection Reagent (Promega, Charbonnières les Bains, France). Forty-eight hours post transfection, 3 ml of supernatant were collected; FBS was added to a final concentration of 10% and clarified by passage through a 0.45 μm filter. The filtrates were aliquoted and stored at -80°C until needed for p24 ELISA assay and infectiousness test on TZM-bl reporter cells.

#### Titration

Titration of the chimeric viruses was performed in TZM-bl cells, permissive for both R5- and X4-tropic viruses as previously described [37], v*ia* the Tat-induced expression of two reporter genes, luciferase and β-galactosidase respectively. The level of infection was determined by measuring luminescence activity (TriStar LB941, Berthold Technologies, Bad Wildbad, Germany) using non-infected cells as a negative control, and a laboratory virus (HIV-1 Lai) and a primary isolate (SF162) as positive controls. In practice, TZM-bl cells were incubated with 50 μl of culture supernatant containing the chimeric virus and with 20 ng/ml of DEAE-Dextran (Sigma-Aldrich, Saint-Quentin Fallavier, France) for 48 h. Luciferase substrate (Bright-Glo^TM^ Luciferase Assay System, Promega) was then added to the cells for 5 min before measurement of luminescence. The luminescence signal was acquired during 5 sec for each well after a brief shake of the plate, using the TriStar LB941 apparatus. Results were expressed in Relative Luminescence Unit (RLU). Four to seven transfections were performed for each plasmid; each titration in TZM-bl was done in duplicate.

#### Visualization by CM

We first wanted to check whether the viral constructs expressing green fluorescent proteins could be used to locate them within cells during infection experiments. To this end, cell-free particles were seeded on glass cover-slips in 6-well plates for 3h, washed and fixed in 4% paraformaldehyde-PBS for 2 h. Twenty μg/ml of the primary HIV-1 Gag p24 goat polyclonal antibody (LS-C129839, LSBio, Seattle, USA) in PBS containing 10% FBS was added for 1 h. After several washing steps, slides were finally incubated with 2.7 μg/ml of the secondary Alexa Fluor™ 555 donkey anti-goat antibody (Invitrogen, Oregon, USA) for 2 h in PBS containing 10% FBS. After several washing steps, coverslips were mounted using Fluoprep mounting medium (Biomérieux, Marcy l’Etoile, France) before visualization by CM.

To check the intracellular location of viral particles expressing green fluorescent proteins, 5x10^6^ HEC-1A cells were cultured for 72h in McCoy medium added with 10% fetal bovine serum in 24-well microplates in the presence of chimeric viruses at the p24 concentration of 200 pg/ml. Cells were then washed, fixed in 4% PFA-PBS for 2 h and incubated for 10 min in 50 mM NH_4_Cl to quench free aldehydes. Infected cells were incubated overnight at 4°C in the dark with a mixture of mouse anti-human ZO-1 monoclonal antibody (BD Biosciences, Le Pont de Claix, France) and HIV-1 Gag p24 goat polyclonal antibody (LSBio). After several washing steps, cells were then incubated with Alexa Fluor™ 555 goat anti-mouse IgG (H+L) and Alexa Fluor™ 555 donkey anti-goat antibodies (Invitrogen). After several washing steps, coverslips were mounted using Fluoprep mounting medium before visualization by CM.

### Infection of HEC-1A cells by X4- and R5-tropic HIV-1 strains

#### Visualization by CM

Cells were seeded on glass cover-slips in 6-well plates for 3 days, followed by incubation with chimeric viruses expressing green fluorescent proteins, during various times (3, 5, 8, 15 and 24 h). Cells were washed and fixed in 4% PFA-PBS for 2 to 6 h and then incubated for 10 min in 50 mM NH_4_Cl to quench free aldehydes. After an incubation step of 20 min, cells were incubated overnight at 4°C in the dark with mouse anti-human ZO-1 monoclonal antibody (BD Biosciences), diluted in PBS containing 10% FBS. Following washing steps, cells were incubated with Alexa Fluor™ 555 donkey anti-mouse IgG (H+L) antibody (Invitrogen), for 2 h in PBS containing 10% FBS. After several washing steps, coverslips were mounted using Fluoprep mounting medium. Three infection experiments were performed per construct.

#### Analysis of infection by real-time PCR

HEC-1A cells were cultured in 12-well plates as described above. When a confluence of 80% was obtained, cells were incubated for 24 h with different HIV-1 strains (Lai, BaL, 92US660 or 92US029), using 200 pg of p24 for each strain. The presence of intracellular particles and reverse transcription activity was evaluated by quantifying HIV RNA and HIV DNA, respectively, using real-time PCR assay as described above.

The use of CXCR4 and CCR5 co-receptors for HIV entry was verified by incubating the cells with 100 ng/ml of rhSDF-1α (CXCL12) or rhRANTES (Peprotech, Rocky Hill, NJ, USA) 2 h before adding CXCR4- or CCR5-tropic strains respectively in cell culture medium. The effect of azidothymidine (AZT) (10 μmol/l, GlaxoSmithKline, Marly-le-Roi, France) on HIV reverse transcription was studied under the same conditions.

In some experiments, cells infected with HIV for 24 h were incubated with proinflammatory cytokines rhTNF-α (10 ng/ml) and rhIL-1β (25 ng/ml) (Peprotech) for an additional 24 h, before quantifying intracellular and extracellular HIV RNA and DNA as well as p24 production in cell supernatant.

#### Experiments of HIV-1 transmission from HEC-1A cells to PBMCs

HEC-1A cells were cultured in semi-permeable inserts with 0.3 μm pores (24 wells per plate) until a tight monolayer was obtained (controlled by trans-epithelial electrical resistance values of 450–600 ohm.cm^2^). They were then incubated for 24 h with chimeric viruses (pBrNL4.3-HXB2-dsRedExpress and/or pBrNL4.3-Bal-eGFP) at the p24 concentration of 200 pg/ml. After 3 washing steps with PBS, infected HEC-1A inserts were deposited into wells containing activated PBMCs (15x10^6^ PBMC per well) and incubated for 24 h. PBMCs were then recovered, washed, fixed and incubated with a mouse anti-human CD26 antibody (Santa Cruz Biotechnology Inc, Heidelberg, Germany) diluted in PBS containing 10% FBS overnight in the dark. After several washing steps, cells were then incubated for 2 h in PBS containing 10% FCS with relevant anti-mouse secondary antibodies (Invitrogen), depending on the fluorochrome expressed by the chimeric virus used: Alexa Fluor™ 488 donkey anti-mouse IgG (H+L) (green) in combination with pBrNL4.3-dsRedExpress, Alexa Fluor™ 555 goat anti-mouse IgG (H+L) (red) in combination with pBrNL4.3-eGFP, or Alexa Fluor™ 647 donkey anti-mouse IgG (H+L) (blue) in combination with both constructs. After several washing steps, coverslips were mounted using Fluoprep mounting medium before visualization by CM. Despite the fact that CD26 is not specific of PBMCs, the use of this marker was justified by the availability in the laboratory of anti-CD26 antibodies with three different labels; as the analysis was performed morphologically and PBMCs were the exclusive cell population present at the basal side of the filter, the risk of confusion with epithelial cells was negligible.

## Results

### Properties of chimeric viruses

Chimeric vectors were transfected in HEK-293 cells. Three chimeric viruses were produced, two expressing green proteins (pBrNL4.3-HXB2-eGFP and pBrNL4.3-BaL-eGFP) and one expressing red proteins (pBrNL4.3-HXB2-dsRedExpress). As shown in Fig 1B, p24 mean values of 256 (± 91), 180 (± 40) and 325 (± 64) pg/ml were obtained for each of these viruses, respectively.

These recombinant viruses were then tested for their infectiousness using TZM-bl reporter cells. Median [min-max] RLU values of 1.38 x 10^5^ [3.1 x 10^4^–2.2 x10^6^], 2.7 x10^5^ [2.2 x10^4^–5.9 x 10^6^] and 2.0 x10^5^ [2.0 x 10^5^–4.2 x10^5^] were obtained for each of these three viruses, respectively.

### Validation of chimeric viruses as markers of HIV-1 infection

Due to the fact that the chimeric replicative viruses were not directly tagged but were able to express fluorescent proteins, it was important to assure that these viruses, unlike pseudoviruses, could support several cycles of infection and could be used for detecting the presence of viral particles within the epithelial cells.

**Fig 2:**
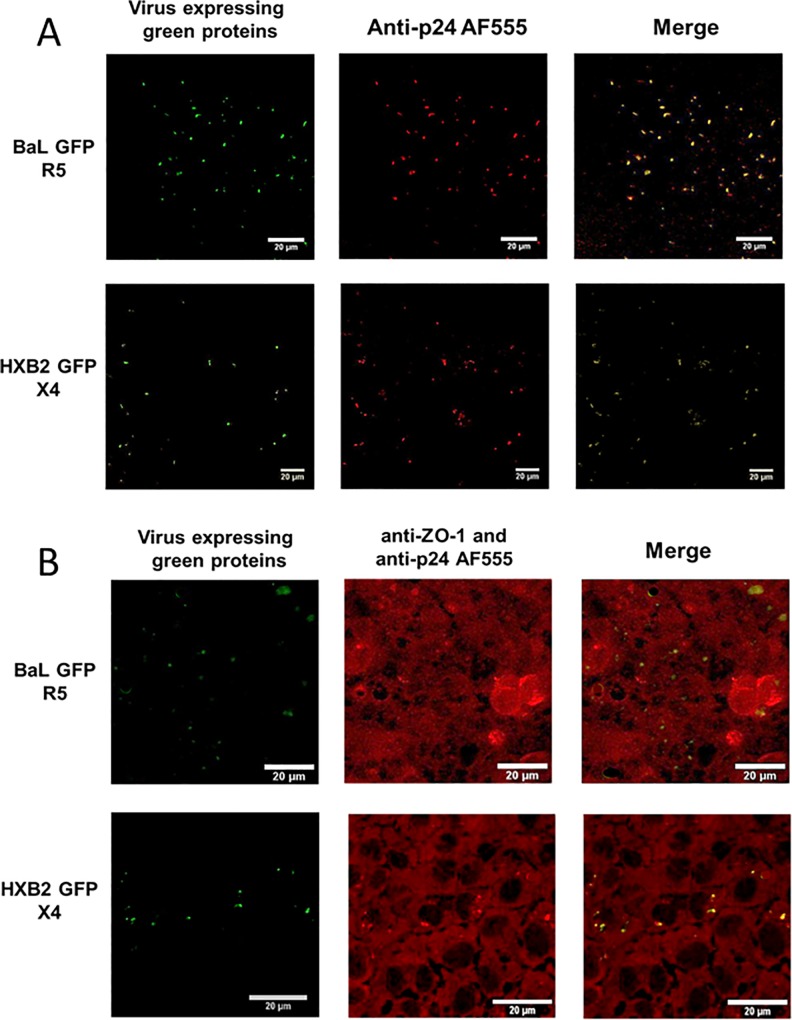
**Visualization by confocal microscopy of pBrNL4.3-BaL-eGFP and pBrNL4.3-HXB2-eGFP constructs using cell-free viruses deposited on glass cover-slips (A) or HEC-1A cells infected by chimeric viruses (B).** The left panels correspond to spontaneous green fluorescence of the respective constructs; the middle panels correspond to p24 (free virus in A) or p24 and ZO-1 (infected HEC-1A cells in B) red immunostaining; the right panels correspond to merged pictures of the two previous ones in order to demonstrate the colocalzation of the signals and to validate the use of these constructs in further experiments.

As shown in Fig 2 for viruses expressing green proteins, the p24 staining co-localized with the eGFP signal in CM for cell-free viruses deposited on glass cover-slips (Fig 2A). Similarly, the p24 and ZO-1 staining co-localized with the eGFP signal in HEC-1A cell cultures infected by chimeric viruses (Fig 2B). These results validated the use of chimeric viruses for locating viral particles within epithelial cells by CM.

### Visualization of infection kinetics by X4- or R5-tropic chimeric viruses in HEC-1A cells

**Fig 3:**
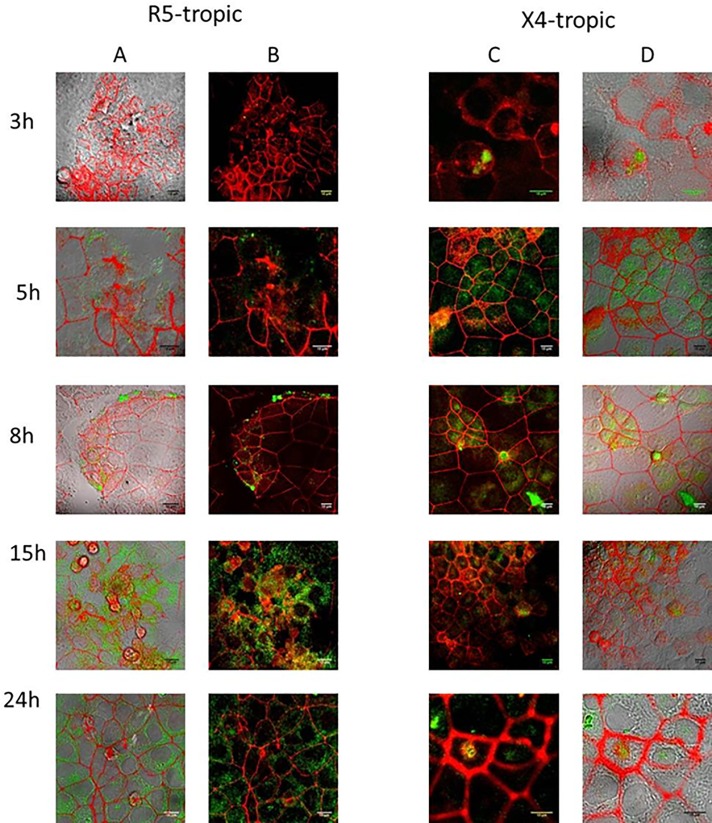
Visualization of kinetics of HEC-1A infection by X4 and R5 chimeric viruses by confocal microscopy. HEC-1A cells were infected for 3, 5, 8, 15 and 24 h by R5-tropic pBrNL4.3-Bal-eGFP chimeric viruses (columns A and B) or X4-tropic pBrNL4.3-HXB2-eGFP chimeric viruses (columns C and D). ZO-1 cellular proteins are colored in red and eGFP signals in green. A and D columns correspond to Differential Interference Contrast (DIC) representations. B and C columns correspond to the green fluorescence channel (green foci correspond to the location of viral particles). Each experiment was done in triplicate, of which a representative example is shown. Scale bars correspond to 10 μm.

Chimeric viruses exhibiting an X4- (pBrNL4.3-HXB2-eGFP) or R5- (pBrNL4.3-BaL-eGFP) tropism were incubated with HEC-1A cells during various times (3, 5, 8, 15 and 24 h). The localization of viral infection was studied by CM (Fig 3 and S1 Fig for enlargement of the images). Interestingly, the X4-tropic strain was localized mainly in the nuclei starting from 8h of contact (Fig 3C and 3D and S1 Fig), whereas the R5-tropic strain exhibited an exclusive cytoplasmic location until 24 h of contact (Fig 3A and 3B and S1 Fig).

To standardize the analysis of the pictures obtained by CM, areas found positive for the presence of chimeric viruses in epithelial cells were specifically selected. The respective proportion of marked cells was quantified at 3, 15 and 24 h post-infection. From 3 to 24 h of contact, the percentage of positive cells slightly increased for the R5-strain whereas it was relatively stable for the X4-strain (data not shown).

### Confirmation of HEC-1A infection by HIV-1 nucleic acid quantification

To verify that the signals observed by CM corresponded to intracellular location of chimeric viruses, HEC-1A cells were infected by Lai or BaL viruses and HIV-1 RNA from infected cells was tested by quantitative real-time PCR assay. A similar amount of intracellular HIV RNA for X4- and R5-tropic viruses was found after 24h of infection (9x10^5^ and 1x10^6^ copies/ml, respectively in one experiment). No HIV RNA was detected in cells treated with rhSDF-1α and incubated with HIV Lai, suggesting that the X4-tropic virus used the CXCR4 co-receptor to enter the cells (data not shown). On the contrary, R5-tropic viruses did not use the CCR5 co-receptor to enter into HEC-1A cells since their incubation with rhRANTES, the natural ligand of CCR5, did not inhibit the viral entry (data not shown).

**Fig 4:**
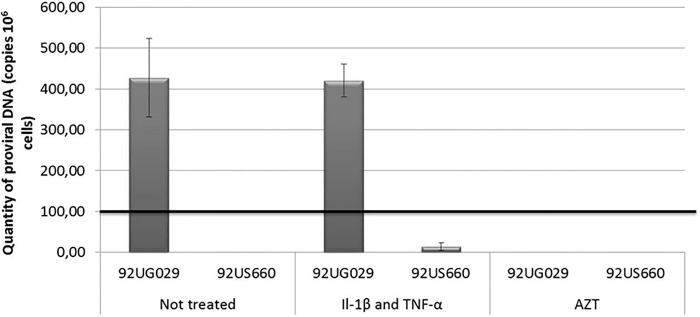
Results of HIV DNA quantification in HEC-1A cells 24 h after infection by X4- or R5-tropic primary isolates (92UG029 and 92US660 strains, respectively). The two right columns correspond to untreated cells. The two central columns correspond to the addition of a mixture of Il-1β (25 ng/ml) and TNF-α (10 ng/ml) pro-inflammatory cytokines 24 h post-infection. The two right columns correspond to the addition of azydothimidine (AZT) 10 μM 2 h before infection. Results are representative of 6 independent experiments; each of them was performed in duplicate as well as the measurement of total HIV DNA.

Fig 4 illustrates the results obtained with HEC-1A cells infected by HIV-1 primary isolates exhibiting X4 or R5 profiles. Whilst both viruses entered the cells, HIV DNA was detected in cells infected by the X4-tropic primary isolate 92UG029 but not in those infected by the R5-tropic primary isolate 92UG660. The incubation of cells with AZT prevented the production of HIV DNA after infection with Lai, suggesting that the genome of X4-tropic virus was reverse-transcribed and could penetrate into the nucleus of HEC-1A cells. Nevertheless, no production of new viral particles was observed in these cells as determined by measuring the quantity of p24 viral proteins in the supernatant of infected HEC-1A cells, 24 and 48 h post infection (data not shown). To check the ability of HIV DNA to be activated, pro-inflammatory cytokines TNF-α and IL-1β were added to infected cells: no change was observed in terms of intracellular HIV DNA concentration (Fig 4), intracellular HIV RNA and quantity of p24 protein in the supernatant of infected cells.

### Transmission of HIV-1 from epithelial cells to PBMCs

**Fig 5:**
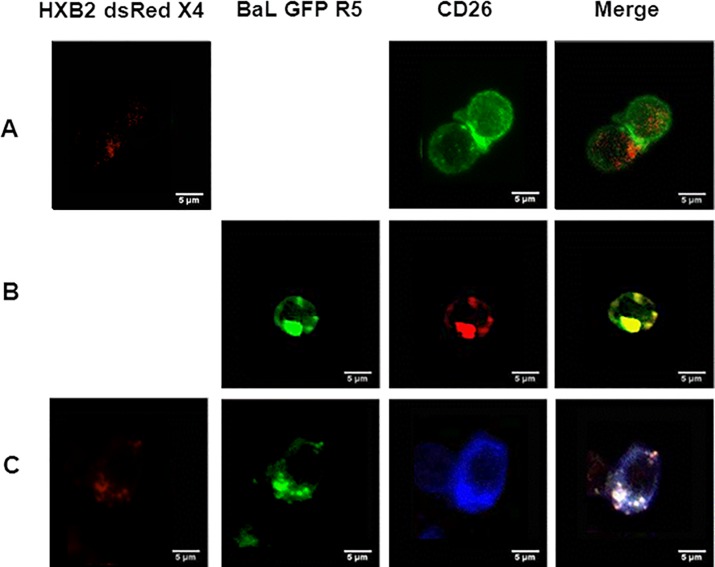
Transmission experiments of HIV from infected HEC-1A cells to uninfected PBMCs by using semi-permeable inserts with 0.3 μm pores. Confluent HEC1-A cells cultured on the upper part of the inserts were infected for 24h with pBrNL4.3-HXB2-dsRedExpress designed as HxB2 dsRed X4 (A), pBrNL4.3-BaL-eGFP designed as BaL GFP R5 (B) or both chimeric viruses (C). After three washing steps, inserts were then deposited into 24-well plates containing activated PBMCs (15x10^6^ cells per well) and incubated for 24h. PBMCs were then recovered, washed and fixed. An anti-CD26 antibody, labeled with Alexa Fluor™ (AF) 488 in A, AF 555 in B and AF 647 in C, was used for staining PBMCs.

The ability of epithelial cells to transmit viral particles to immune permissive cells was checked by first infecting confluent HEC-1A cells in the upper chamber of inserts with R5-tropic (pBrNL4.3-BaL-eGFP), X4-tropic (pBrNL4.3-HXB2-dsRedExpress) or both chimeric viruses (Fig 5). Next, the inserts were washed 3 times at the basal and apical sides and deposited into wells containing activated PBMCs at the basal side. Weak fluorescence was observed by CM in PBMCs incubated in presence of HEC-1A cells infected by the X4-tropic chimeric virus (Fig 5A). In contrast, a clear positive signal was detected in PBMCs associated to HEC-1A cells infected by the R5-tropic chimeric virus (Fig 5B). When HEC-1A cells were concomitantly infected by X4- and R5-tropic chimeric viruses, a strong signal was observed for the green R5 virus whereas only a weak signal was detected for the red X4 virus in PBMCs (Fig 5C).

## Discussion

The aim of the present study was to revise the debated issue of HIV-1 infection in genital epithelial cells by using CM to visualize the fate of X4- or R5-tropic viruses in the HEC-1A cell line, a well-established *in vitro* model of endometrial epithelial cells [29,32]. One of the most important characteristics of epithelial cells is to be polarized with different apical and basal lipid and protein composition and to form selective barriers at the surface of the tissues [38]; the HEC-1A cell line was shown to exhibit this property when cultured on different supports including filters and solid surfaces like glass slides, as previously shown by transepithelial electrical measurements [12].

Visualization of HIV-1 in the female genital mucosa was previously performed by using fluorescent antibodies directed against viral p24 core protein [2]. In other studies, constructs were directly tagged by various fluorescent markers. For example, a fluorescent virus carrying eGFP at the C terminus of the matrix domain of Gag was used to assess specifically the localization and trafficking of Gag protein towards polysynapses in living T cells [17]. Infectious and non-infectious GFP-Gag tagged viral particles were also constructed to establish the role of intestinal DCs in the transportation of HIV-1 particles through the intestinal mucosa and their subsequent transmission to T cells; the same constructs were used in experiments demonstrating that the macrophages present in vagina are permissive to HIV-1 [22]. However, studies dealing with the localization of viral particles in cells and their ability to cross the mucosal barrier are very few. Campbell et al. generated virions dually labeled with S15-mCherry and GFP-Vpr; the loss of the S15-mCherry membrane signal was observed following fusion with the cell membrane, but also the signal of GFP-Vpr, 4 h after cell infection [20]. The infectious, replicative-competent chimeric viruses expressing dsRedExpress or eGFP proteins that were used in the present work were initially developed for studying viral infectivity under different experimental conditions [35,36,39]. In our hands, they were used as cell-free viruses exhibiting an R5- or X4-tropic envelop. As the viruses were not directly tagged, it was important to control that the observed fluorescent signals co-localized with viral particles. We demonstrate herein that the constructs were able to generate a stable post-infection signal both in cell-free and cell-associated models (Fig 2) and could be used further for studying the *in vitro* mucosal transmission of HIV-1. In addition to these experimental results, the use of recombinant viruses exhibiting a reporter gene has been validated earlier for assessing different HIV-1 properties [40–43].

Combining pictures of CM with molecular biology experiments, our study aimed to address the following questions: (i) Can free R5- and X4-tropic particles of HIV-1 be transmitted to epithelial cells from a female genital tract monolayer? (ii) If the answer to this first question is positive, is this infection productive? (iii) Can they further transmit the viruses to immune target cells?

With regard to the first question, our results confirm that R5- and X4-tropic viruses both enter HEC-1A cells [29] but exhibit different kinetics of infection, suggesting that they use different pathways to penetrate and infect these cells. Endocervical HEC-1A cells are known to express at their surface the CXCR4 co-receptor but not the CD4 receptor and the CCR5 co-receptor [29,32]. The present data demonstrate that X4-tropic viruses do use the CXCR4 co-receptor since neither RNA nor DNA was detected in infected cells after treatment by rhSDF-1α, the natural ligand of CXCR4. On the contrary, the blockage of CCR5 by rhRANTES did not alter the penetration of R5-tropic constructs into HEC-1A cells since intra-cytoplasmic particles were observed by CM and HIV RNA could be quantified by RT-PCR. Mathematical analyses of z stacks from CM pictures allowed to state that viral particles were inside the cells and not at their external surface. Furthermore, after penetration into HEC-1A cells, R5-tropic viruses remained localized near the plasma membrane (Fig 3).

Remarkably, no virus production could be measured in the supernatant of HEC-1A cells infected with either X4 or R5 HIV-1 strains, confirming earlier findings [29]. With regard to X4-tropic viruses, our results on detection of intracellular LTR DNA are in accordance with previous studies as well [29,32], suggesting that X4-tropic viruses are sequestrated after infection of HEC-1A and reverse transcription without viral production. Interestingly, our X4 viral constructs were visualized in the nucleus of HEC-1A cells (Fig 3). In a previous study using an amplification of the *alu-gag* region followed by a specific HIV PCR (LTR), we were able to show that DNA from X4 strains were able to be integrated within the cellular genome of different epithelial cell lines including HEC-1A [32]. By contrast to X4-tropic strains, the exclusively cytoplasmic location of R5-tropic particles (Fig 3) together with the absence of detection of intracellular LTR DNA suggest that no reverse transcription occurred after infection of HEC-1A cells by R5-tropic strains.

Following the observation that HEC-1A infection by HIV-1 was not productive, we evaluated the potential ability of the epithelial reservoir to be activated under a pro-inflammatory environment. For this, X4- and R5-infected HEC-1A cells were treated by TNF-α and IL-1β, since these cytokines were found to promote CCL20 secretion by EC [32,44]. We show herein that HIV-1 replication was not enhanced by the presence of pro-inflammatory cytokines for both X4- and R5-tropic strains (Fig 4).

A recent paper conducted on oral and vaginal cell lines originating from multilayered epithelia led to the conclusion that these cells could be infected by cell-free infectious HIV-1 particles but were not productively infected [30]; interestingly, by contrast to our results, R5- and X4-tropic strains were shown to exhibit a similar pattern of infection. The main difference between epithelial monolayers (such as HEC-1A cells) and multilayers (as the cell lines tested in [30]) is the absence of expression of CXCR4 co-receptor on the latter cells, which suggests that the cell-free virions use non-canonical receptors to enter the epithelial cells, as did the R5-tropic viruses in our model. In accordance with our results, studies conducted with primary uterine [45] and gingival [46] epithelial cells have also demonstrated preferential transmission of X4-tropic viruses to these cells, in relation with the presence of CXCR4 and GalCer at the surface of these cells.

Concerning the ability of infected epithelial cells to further transmit HIV-1 to activated PBMCs, our results provide evidence that free R5-tropic particles can be transmitted, whereas the transmission of X4-tropic viruses was much more difficult. These findings are in accordance with the preferential transmission of R5-tropic strains during genital primary HIV-1 infection (for reviews see [47,48]) but also with recent findings suggesting that X4-tropic strains can exceptionally be transmitted at this stage [49]. It is interesting to notice that Kohli et al. also concluded to the ability of cell lines from multilayered epithelia not productively infected by HIV to transmit free particles to competent cells [30].

As a whole, it can be hypothesized from this study and others [29,32,47–53] that X4-tropic viruses penetrate into HEC-1A cells by using the CXCR4 co-receptor through membrane fusion, and reverse transcribe their genome without completion of a productive replication cycle, whereas R5-tropic viral particles enter these cells probably by using alternative receptors, which could lead to the transcytosis of viruses through the cytoplasm of monolayered epithelial cells, without starting a replication cycle in these cells. Both R5- and to a lesser extent X4-tropic strains can be transmitted to permissive immune-competent cells. However, this mode of infection is probably not very efficient as shown by our inability to document the presence of infectious virus by p24 testing or amplification of HIV RNA.

Most reports dedicated to the study of virus-cell interactions at the mucosal level have used laboratory or blood-derived strains that are poorly representative of the seminal reservoir. Indeed, viruses found in the genital tract can exhibit distinct genetic characteristics, especially in the C2/V5 region of gp120 [54–58]. It would appear crucial to use seminal strains for studying heterosexual transmission in *in vitro* models, at least to confirm results obtained with commonly used laboratory strains. In this work, we attempted to incorporate in our constructs a complete gp160 protein amplified from seminal plasma. Unfortunately, we were unable to obtain a stable replicative chimeric virus (data not shown). It may be due to the amplification of a defective particle mutated on the *env* gene that was unable to enter or replicate into the cells. It cannot also be excluded that some primary sequences may be not functional in the context of the vector used in this study.

In conclusion, the approach described herein may contribute to elucidate the putative role of epithelial monolayers (as that found in the endocervix) in the selection of R5-tropic viruses during heterosexual transmission. In the present study we focused on the transmission of cell-free particles through a monolayered genital cell line. Further work is needed to evaluate whether these results can be extended to more complex models of reconstructed mucosa more prone to mimic the *in vivo* heterosexual transmission, including cell multilayers, primary cell lines or ex vivo explants, containing mucosal immune cells able to support HIV replication (Langerhans cells, T lymphocytes, macrophages) and using cell-associated viruses from seminal origin as the source of infection. This morphological approach would also constitute a useful tool to evaluate the efficacy of various compounds such as neutralizing antibodies or microbicides to block HIV transmission in the genital mucosa.

## Supporting Information

S1 Fig Enlarged images of the panels depicted in Fig 3 of the printed version. Each slide corresponds to a different time post-infection. The R5-tropic strain of HIV-1 is shown on the left and the X4-tropic strain on the right. Large squares are confocal microscopy pictures (with white arrows indicating the presence of green-labeled viruses within the cells). Small squares correspond to Differential Interference Contrast (DIC) representations.
(PDF)
