# Supplementary material for: Visualization of X4- and R5-Tropic HIV-1 Viruses Expressing Fluorescent Proteins in Human Endometrial Cells: Application to Tropism Study
Source: PLoS One. 2017 Jan 6;12(1):e0169453. doi: 10.1371/journal.pone.0169453 (PMC5218496; doi:10.1371/journal.pone.0169453)

S1 Fig. Enlarged images of the panels depicted in Figure 3 of the printed version. Each slide corresponds to a different time post-infection. The R5-tropic strain of HIV-1 is shown on the left and the X4-tropic strain on the right. Large squares are confocal microscopy pictures (with white arrows indicating the presence of green-labeled viruses within the cells). Small squares correspond to Differential Interference Contrast (DIC) representations.

3H post-infection

X4

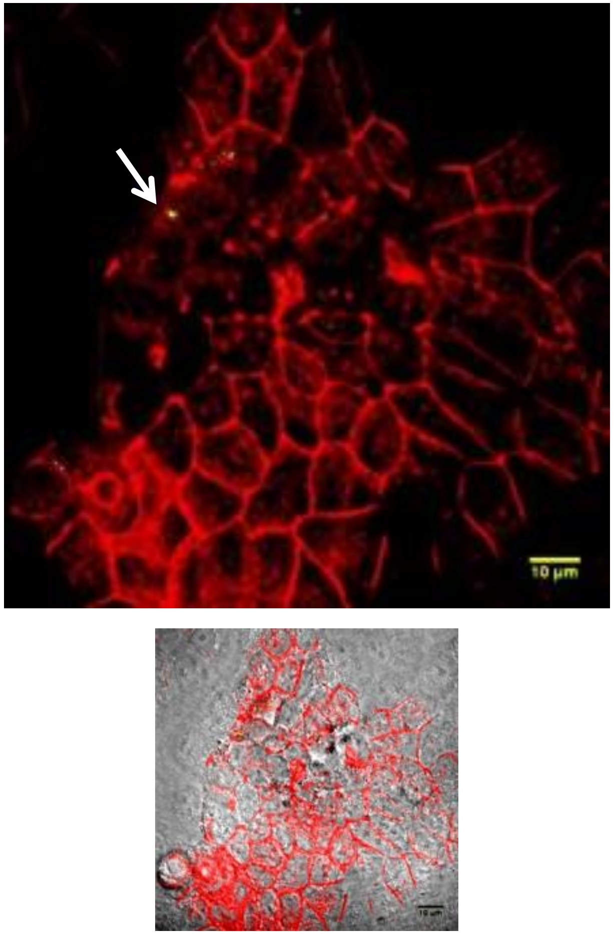

R5

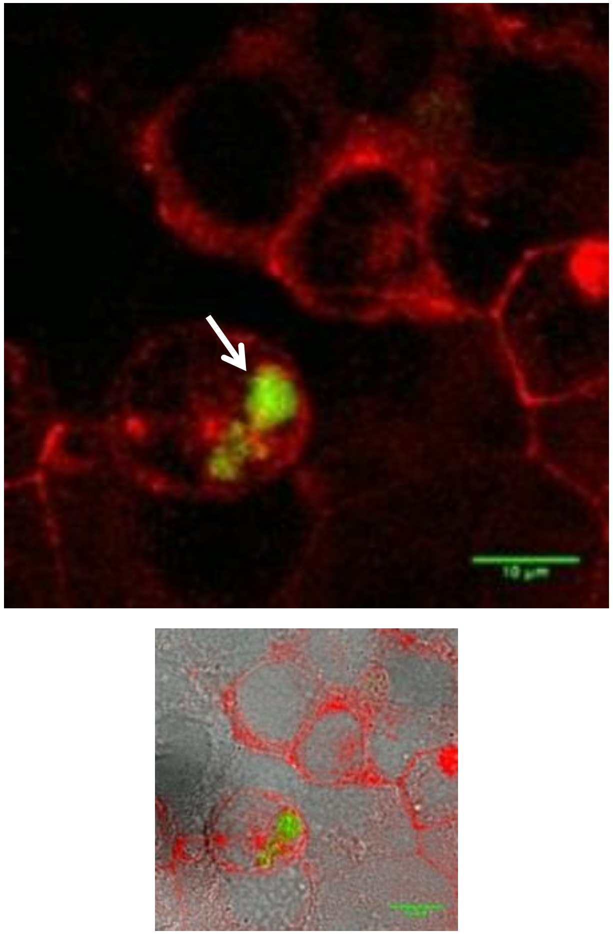

5H post-infection

X4

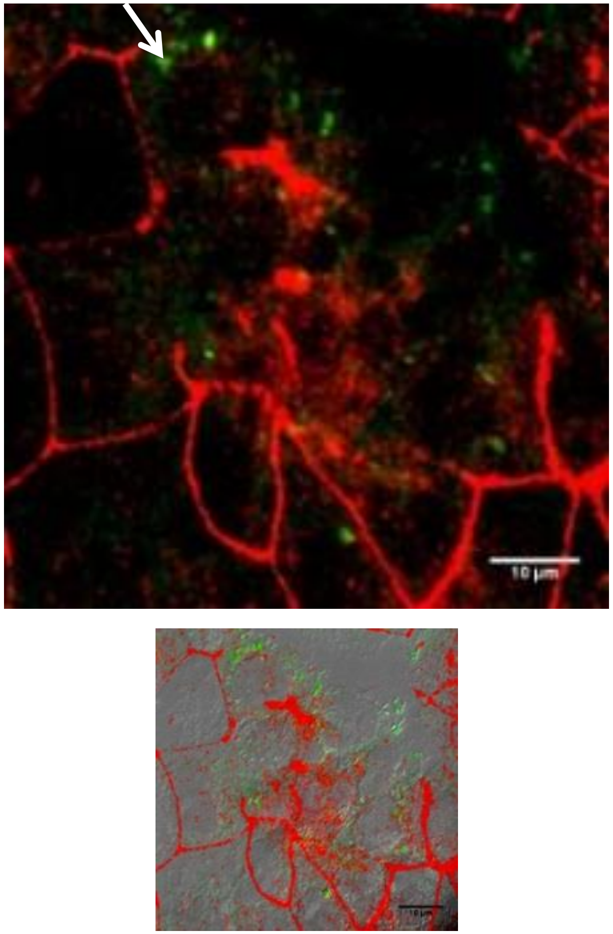

R5

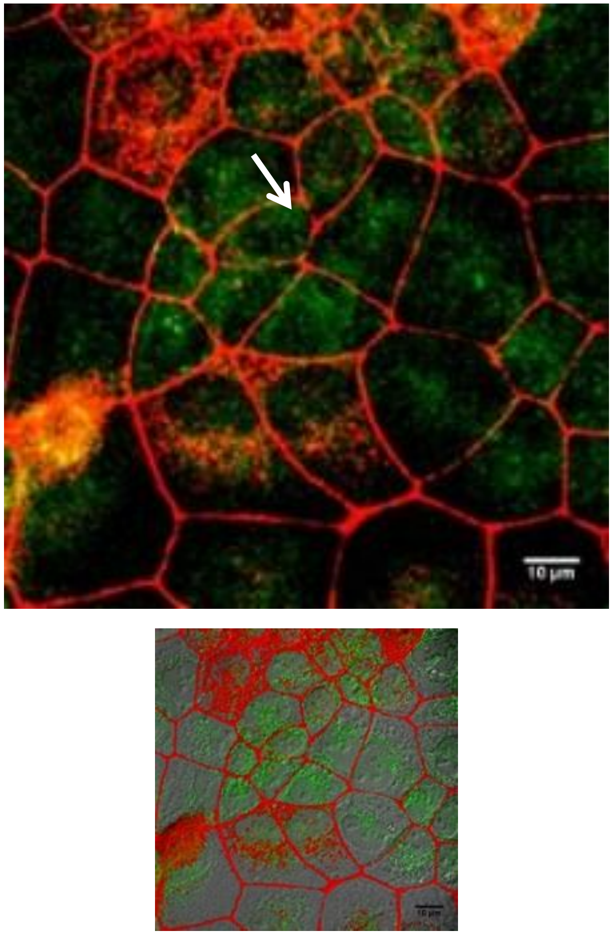

8H post-infection

X4

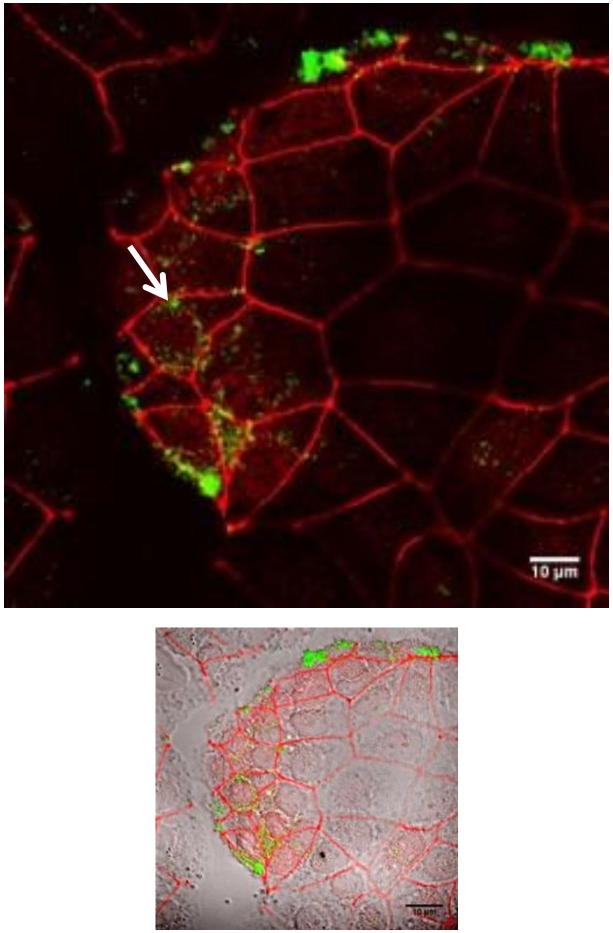

R5

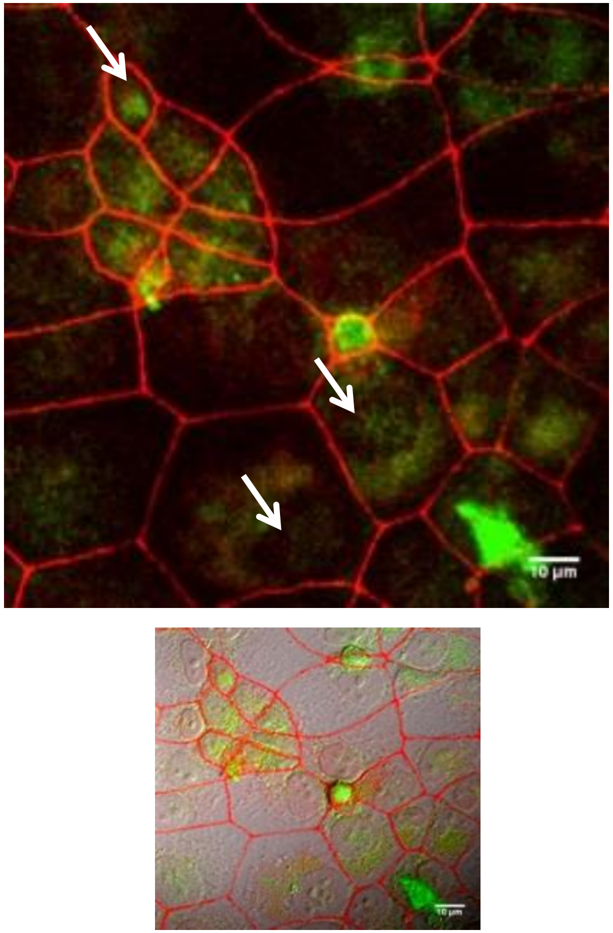

15H post-infection

X4

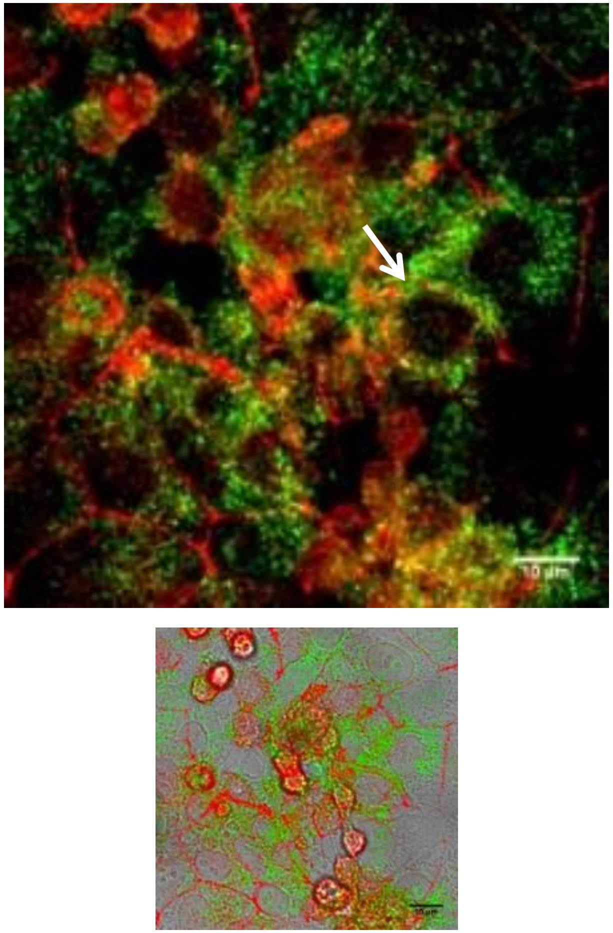

R5

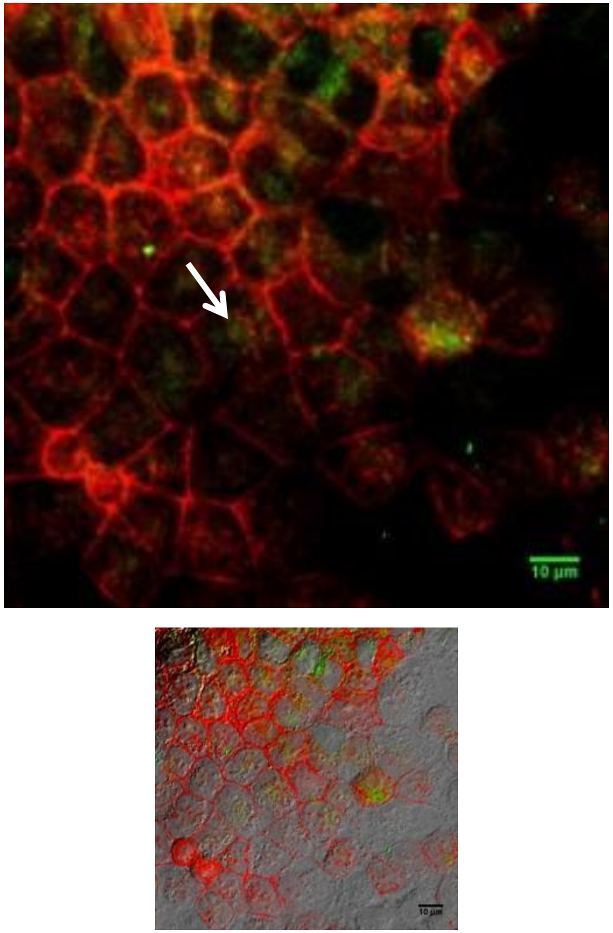

24H post-infection

X4

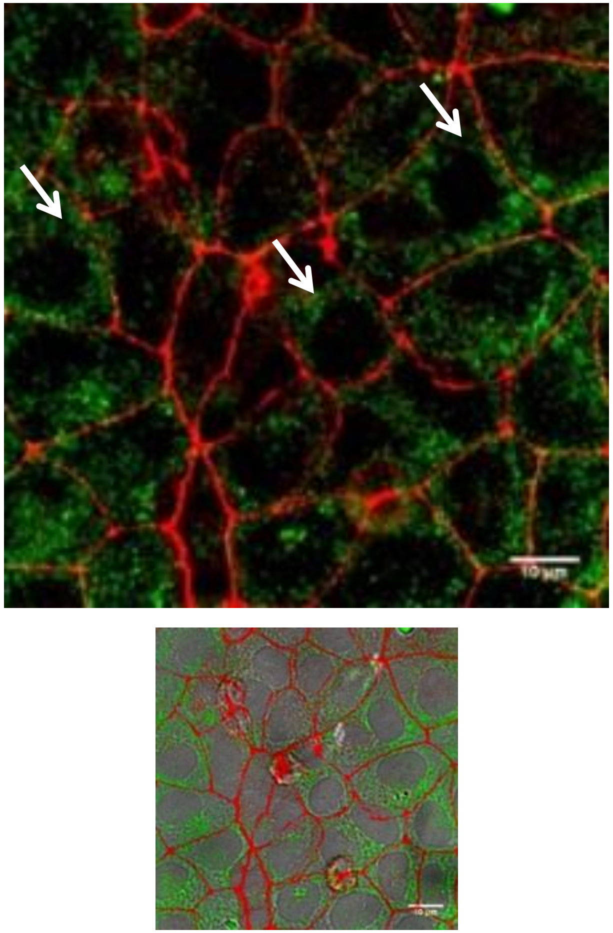

R5

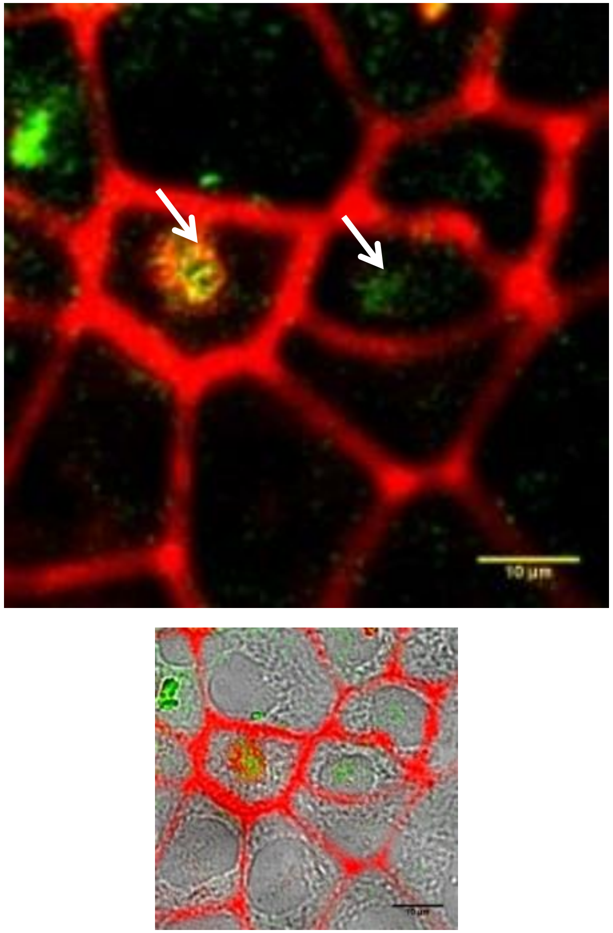

Supplement: S1 Fig — Each slide corresponds to a different time post-infection. The R5-tropic strain of HIV-1 is shown on the left and the X4-tropic strain on the right. Large squares are confocal microscopy pictures (with white arrows indicating the presence of green-labeled viruses within the cells). Small squares correspond to Differential Interference Contrast (DIC) representations. (PDF) [file pone.0169453.s001.pdf]
